# Tissue-Based Metabolomic Profiling of Endometrial Cancer and Hyperplasia

**DOI:** 10.3390/metabo15070458

**Published:** 2025-07-05

**Authors:** Khalid Akkour, Afshan Masood, Maha Al Mogren, Reem H. AlMalki, Assim A. Alfadda, Salini Scaria Joy, Ali Bassi, Hani Alhalal, Maria Arafah, Othman Mahmoud Othman, Hadeel Mohammad Awwad, Anas M. Abdel Rahman, Hicham Benabdelkamel

**Affiliations:** 1 Obstetrics and Gynecology Department, College of Medicine, King Saud University, Riyadh 11461, Saudi Arabia; kakkour@ksu.edu.sa (K.A.); abassi@ksu.edu.sa (A.B.); 2 Proteomics Resource Unit, Obesity Research Center, College of Medicine, King Saud University, Riyadh 11461, Saudi Arabia; afsmasood@ksu.edu.sa (A.M.); aalfadda@ksu.edu.sa (A.A.A.); oalothman@ksu.edu.sa (O.M.O.);; 3 Metabolomics Section, Personalized Medicine Laboratory Department (PMLD), Genome Medicine Center of Excellence (GMCoE), King Faisal Specialist Hospital and Research Centre (KFSHRC), Riyadh 11211, Saudi Arabia; mmogren@kfshrc.edu.sa (M.A.M.); rgalmalki@kfshrc.edu.sa (R.H.A.); 4 Department of Medicine, College of Medicine, King Saud University, Riyadh 11461, Saudi Arabia; 5 Strategic Center for Diabetes Research, College of Medicine, King Saud University, Riyadh 11461, Saudi Arabia; sjoy@ksu.edu.sa; 6 Obstetrics and Gynecology Department, King Saud University Medical City, King Saud University, Riyadh 11461, Saudi Arabia; halhalal@ksu.edu.sa; 7 Department of Pathology, College of Medicine, King Saud University Medical City, King Saud University, Riyadh 11461, Saudi Arabia; mariaarafah@ksu.edu.sa; 8 Department of Biochemistry and Molecular Medicine, College of Medicine, Alfaisal University, Riyadh 11533, Saudi Arabia

**Keywords:** endometrial cancer, hyperplasia (HY), metabolomics, LC-HRMS, biomarker

## Abstract

**Background**: Endometrial cancer (EC) is the sixth most common cancer among women globally, with an estimated 420,000 new cases diagnosed annually. **Methods**: This study comprised patients with endometrial cancer (EC) (n = 17), hyperplasia (HY) (n = 17), and controls (CO) (n = 20). Tissue was collected from the endometrium of all 54 patients, including patients with HY, EC, and CO, who underwent total hysterectomy. EC and HY diagnoses were confirmed based on histological examination. Untargeted metabolomics profiling was conducted using LC-HRMS. The partial least squares discriminant analysis (PLS-DA) and orthogonal partial least squares discriminant analysis (OPLS-DA) models were used for univariate and multivariate statistical analysis. The fitness of the model (R2Y) and predictive ability (Q2) were used to create OPLS-DA models. ROC analysis was carried out, followed by network analysis using Ingenuity Pathway Analysis. **Results**: The top metabolites that can discriminate EC and HY from CO were identified. This revealed a decrease in the levels of the lipid species, specifically phosphatidic acid (PA) (PA (14:1/14:0), PA(10:0/17:0), PA(18:1-O(12,13)/12:0)), PG(a-13:0/a-13:0), ganglioside GA1 (d18:1/18:1), PS(14:1/14:0), TG(20:0/18:4/14:1), and CDP-DG(PGF2alpha/18:2), while the levels of 3-Dehydro-L-gulonate, Uridine diphosphate-N-acetylglucosamine, ganglioside GT2 (d18:1/14:0), gamma-glutamyl glutamic acid and oxidized glutathione were increased in cases of EC and HY as compared to CO. Bioinformatics analysis, specifically using Ingenuity Pathway Analysis (IPA), revealed distinct pathway enrichments for EC and HY. For EC, the most highly scored pathways were associated with cell-to-cell signaling and interaction, skeletal and muscular system development and function, and small-molecule biochemistry. In contrast, HY cases showed the highest scoring pathways related to inflammatory disease, inflammatory response, and organismal injury and abnormalities. **Conclusions**: Developing sensitive biomarkers could improve diagnosis and guide treatment decisions, particularly in identifying which patients with HY may safely avoid hysterectomy and be managed with hormonal therapy.

## 1. Introduction

Endometrial cancer (EC) is the sixth most common cancer among women globally, with an estimated 420,000 new cases diagnosed annually. Its incidence is highest in North America and Northern Europe and lowest in Southeast Asia and Africa. EC represents about 90% of all uterine cancers, predominantly affecting postmenopausal women, with an average age of onset around 60 years. EC is characterized by significant biological and clinical heterogeneity from distinct histopathological, molecular, and clinical features. EC is known to develop in patients with genetic mutations and due to hormonal changes, especially those related to estrogens. It is classified into EC’s two major histopathological subtypes based on the Bokhman dualistic model: Type I (estrogen-dependent, which accounts for 80–90% of cases) and Type II (estrogen-independent, which accounts for 10–20% of cases), which are high-grade and clinically aggressive. Preceding the development of EC, there is a stage wherein there is a non-invasive abnormal proliferation of the endometrial lining of the uterus termed endometrial hyperplasia (HY), which is generally considered a precursor for EC [1]. Women with HY are known to carry a high risk of concomitant EC, where 28% are known to progress to EC [2,3,4]. The severity of HY influences clinical management, and if identified early, the rate of progression to EC diminishes by three- to five-fold with hormonal therapy [5]. The clinical presentation of HY and EC is similar, with patients presenting with abnormal uterine bleeding, which is also seen in other benign conditions, making the diagnosis difficult [3,6]. Molecular and tumor microenvironment differences are observed between hyperplasia endometrial biopsies found to have concurrent endometrial cancer in 50% of cases of final hysterectomy specimens and those not [7]. The histopathology does not adequately describe a tumor’s molecular profile. Consequently, more than 20% of ECs initially classified as non-aggressive actually progress to aggressive, metastatic disease. Currently, diagnosing these cancers is difficult because it requires invasive procedures, including a transvaginal ultrasound scan, an outpatient hysteroscopy, and an endometrial biopsy, followed by histopathological analysis. This may reflect occult carcinomas that might remain undetected during the initial biopsy year, only to be diagnosed later, between years two and four. There is a strong need to differentiate among these conditions as early diagnosis of EC improves the prognosis and the survival rate of patients. Understanding the heterogeneity of EC is critical for improving diagnostic accuracy, guiding effective treatment, and developing precision medicine approaches.

While biofluids such as blood and urine are commonly used for metabolomic studies due to their non-invasive collection methods, tissue samples remain the most direct representation of the tumor microenvironment and capture alterations in the cells and their metabolic pathways that are directly reflective of tumor behavior. Metabolomics, the comprehensive analysis of small molecules (metabolites) within biological systems, provides a unique lens for understanding the metabolic changes that occur during the progression of diseases such as EC. Unlike genomics or proteomics, metabolomics offers a real-time snapshot of physiological and pathological states, making it particularly valuable for identifying biomarkers associated with cancer onset, progression, and therapeutic response. Metabolomic profiling can identify early metabolic changes that precede malignant transformation by analyzing pre-cancerous tissues, such as those with hyperplasia. Recent studies, including ours in proteomics and metabolomics, have identified several metabolites and pathways altered in EC, such as an increase in glycolysis and glutaminolysis that leads to the accumulation of lactate and glutamate in tumor tissues and biofluids [8,9,10,11]. Altered lipid metabolism, particularly involving phospholipids and sphingolipids, has also been implicated in EC, correlating with tumor progression and poor prognosis. Amino acids, such as branched-chain amino acids and tryptophan, have emerged as potential biomarkers due to their involvement in tumorigenic pathways [12]. Existing metabolomic analyses have predominantly focused on comparing cancerous tissues with healthy controls, leaving out crucial insights into the intermediate stages of disease progression. Addressing this gap is critical for understanding the early molecular changes associated with EC and developing targeted diagnostic and potential therapeutic strategies. Despite these advancements, few studies have systematically investigated the differences in metabolic profiles of patients with HY and established EC.

The present study aims to utilize untargeted metabolomic profiling of tissue samples from EC and HY in comparison to CO. This study aims to analyze metabolic profiles to fill a crucial gap in understanding the metabolic basis of endometrial cancer (EC) by identifying specific metabolic variables that might increase malignancy risk, which could significantly benefit clinical practice. Advanced analytical platforms such as metabolomics and bioinformatics analysis are employed to uncover key metabolic alterations that can facilitate early detection and personalized treatment of EC.

## 2. Materials and Methods

### 2.1. Population and Study Design

The research methods and procedures were approved by the King Saud University College of Medicine’s institutional review board (IRB number: E-193622), and all participants provided their written consent. This study involved 17 patients with endometrial cancer (EC) (average age 62 ± 9), 17 with hyperplasia (HY) (average age 61 ± 5), and 20 controls (CO) (average age 59 ± 4) recruited from King Khalid University Hospital (KKUH) in Saudi Arabia between May 2012 and November 2022, (Supplementary Materials S1, Table S1). Histological examination confirmed the diagnoses of EC and HY. Exclusion criteria included women who were fertile or had a history of cancer at any other site. During the total hysterectomies, about 100 mg of endometrial tissue was obtained from each of the 54 participants, encompassing the HY, EC, and CO groups. Subsequently, frozen tissue sections underwent histopathological examination (Supplementary Materials S1; Table S1), and fresh tissue samples were promptly snap-frozen in liquid nitrogen for storage at −80 °C until further analysis.

### 2.2. Metabolite Extraction

Tissue metabolites were extracted by the modified protocol from a previous study [13]. Then, 100 mg of tissue was weighed and cut into small pieces. Tissues were transferred into a clean 1.5 mL cryotube with a flat bottom and the tissue was mixed with 40 μL of 70% ice-cold methanol and then homogenized with a homogenizer (T25 digital ULTRA TURRAX homogenizer (IKA-Werke GmbH & Co. KG, Staufen, Germany)). Following this, 40 µL of chloroform and 20 µL of water were added to the tissue and homogenized. The suspension was agitated at room temperature for one hour, followed by sonication using a Microsonicator Q700 (Qsonica Sonicators, Newtown, CT, USA) with a 30% pulse for two 1 min intervals separated by a 1 min pause, chilled at −20 °C for an hour and then centrifuged at 4 °C at 12,000× *g* for 15 min. The liquid supernatants were carefully transferred to Eppendorf tubes, evaporated using a vacuum evaporator (Eppendorf Concentrator plus^TM^; Hamburg, Germany), and stored at −20 °C. Prior to analysis, the dried residue was reconstituted in 100 μL of a 1:1 (*v*/*v*) acetonitrile/water solution, thoroughly vortexed, and then centrifuged again at 4 °C at 12,000× *g* for 15 min. The final supernatant was then collected for LC-MS analysis.

### 2.3. Metabolite Analysis

Untargeted metabolomics profiling was conducted using LC-HRMS, following the established procedures [14]. A Waters ACQUITY UPLC system, coupled with a Xevo G2-S QTOF mass spectrometer featuring an electrospray ionization (ESI) source, was employed to acquire metabolite data in both positive (ESI+) and negative (ESI−) ionization modes. Metabolite separation was achieved using an ACQUITY UPLC HSS C18 2.1 × 100 mm 1.7 µm column (Waters Ltd., Elstree, UK) with a specific gradient elution (0–16 min 95–5% A, 16–19 min 5% A, 19–20 min 5–95% A, 20–22 min 5–95% A) of mobile phases A and B, applied at a 300 µL/min flow rate. The MS conditions included a 150 °C source temperature, 500 °C (ESI+) or 140 °C (ESI−) desolvation temperature, capillary voltages of 3.20 kV (ESI+) or 3 kV (ESI−), a 40 V cone voltage, 800.0 L/h desolvation gas flow, and 50 L/h cone gas flow. For the MS analysis, the MSE mode was utilized with 10 V for low collision energy and 50 V for high functions, with calibration performed using sodium formate across the 100–1200 Da range in both ionization modes. Data-independent acquisition (DIA) was performed using a Masslynx™ V4.1 workstation (Waters Inc., Milford, MA, USA) operating in continuum mode.

### 2.4. Data Handling and Processing

The raw mass spectrometry (MS) data underwent processing using a standard pipeline, alignment based on mass-to-charge ratio (m/z) and ion signal retention time; peak picking and signal filtering based on peak quality, which were implemented with Progenesis QI v.3.0 software (Waters Technologies, Milford, MA, USA) MetaboAnalyst v. 5.0 (McGill University, Montreal, QC, Canada) (http://www.metaboanalyst.ca, accessed on 19 November 2024), were used for multivariate statistical analysis after undergoing median normalization, pareto scaling, and log transformation [15]. Both partial least squares discriminant analysis (PLS-DA) and orthogonal partial least squares discriminant analysis (OPLS-DA) models were generated. The OPLS-DA models were then evaluated based on their fitness (R2Y) and predictive ability (Q2) values. Mass Profiler Professional software version.15.1(Agilent, Santa Clara, CA, USA) was employed for univariate analysis to compare groups, and a one-way ANOVA with Tukey’s post hoc test (no correction, *p* ≤ 0.05) was performed. Significant features were annotated by matching their accurate precursor mass, fragmentation pattern, and isotopic distribution to entries in the Human Metabolome Database (HMDB) [16]. The final list was prepared manually by removing exogenous compounds, including drugs and food additives.

### 2.5. Bioinformatic Analysis

Bioinformatic analysis was caried out using metaboanalyst and Ingenuity Pathway Analysis (IPA) complementarily to ensure rigorous and biologically contextualized interpretation of our data. Metaboanalyst was used to provide statistically robust and topology-aware pathway analysis based on the KEGG database, while IPA was used to provide a more comprehensive, curated knowledge base that uses literature-derived biological networks, and deeper mechanistic insights and functional interpretations. IPA was utilized to investigate the biological functions and interactions of metabolites that exhibited differential expression between EC and control samples, as well as between HY and control samples. The software automatically links the IDs to information within our expert-reviewed Intelligence Knowledge Base, which aggregates data from all published scientific literature. By comparing the experimental data’s expression patterns to established biological networks, the software pinpoints the key activities and pathways significantly linked to the provided metabolite list.

## 3. Results

### 3.1. Mass Ion Detection and Metabolite Identification

A total of 19,146 mass ion features (12323 positive and 6823 negative) were detected in ionization mode (Supplementary Materials S1; Table S2). After several filtration processes, such as alignment, peak picking, and missing value removal, all samples were applied to a filter with a cut-off percentage >80 using Mass Profiler Professional (MPP) software, wherein the metabolites that exceeded 80% of the total samples were excluded from the analysis. This filtration is based on the detection frequency. Statistical analysis (*p* ≤ 0.05 and FC ≥ 1.5) of the remaining 6618 features identified 189 metabolites exhibiting significant dysregulation (Supplementary Materials S1; Table S3). Out of 189 metabolites, only 136 received annotations from The Human Metabolome Database (HMDB), KEGG, MassBank, LipidMAPS, and METLIN MS/MS databases (Supplementary Materials S1; Table S4). Exogenous metabolites (i.e., drugs, drug metabolites, environmental exposures, etc.) were excluded, and 63 endogenous metabolites were identified in both groups, with 30 upregulated and 33 downregulated (Supplementary Materials S1; Table S5). The profile of 30 upregulated metabolites in EC compared to hyperplasia and control after performing a fold change of 2 is shown in Supplementary Materials S2, Figure S1. A profile of 33 downregulated metabolites in EC compared to hyperplasia and control after performing a fold change of 2 is shown in Supplementary Materials S2, Figure S2.

### 3.2. Overview of the Three Study Groups (CO, HY, and EC)

**Figure 1:**
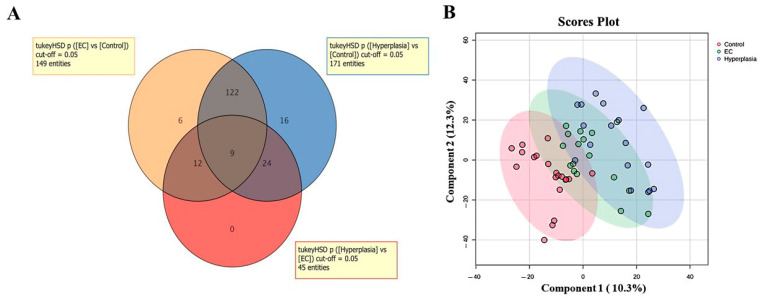
Identified metabolite features across groups. (**A**) The Venn diagram illustrates significant differences in metabolite features among three groups (endometrial cancer (EC) vs. Hyperplasia, endometrial cancer (EC) vs. Control, and Hyperplasia vs. Control) as determined by a one-way ANOVA with Tukey’s post hoc test, FDR *p* < 0.05. (**B**) Partial least squares discriminant analysis (PLS-DA) reveals a partial separation between these three groups.

The overlap of significantly altered ions between the groups was employed using a Venn diagram, and in the three datasets, a total of 365 metabolites were dysregulated (Figure 1A). Furthermore, a PLS-DA model (Figure 1B) was generated to assess sample clustering, group separation, and identify potential outliers within the datasets.

### 3.3. Metabolomic Profiling Between EC and CO

**Figure 2:**
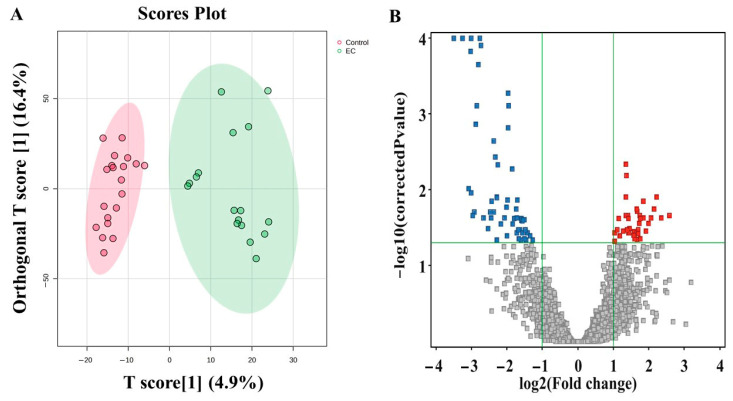
Comparing metabolomic profiles of the endometrial cancer (EC) and Control groups. (**A**) The OPLS-DA score plot effectively separates the endometrial cancer (EC) group from the Control group. The model’s performance was evaluated, yielding a strong fit (R2Y = 0.902) and a Q2 value of 0.51, with these values being found in a larger dataset (n = 100). (**B**) The volcano plot shows significantly dysregulated metabolites in EC compared to the Control group.

Figure 2 represents the metabolites that differentiated the endometrial cancer (EC) from the control (CO) group. The OPLS-DA plot demonstrates a clear separation of tissue metabolites between the EC and CO groups (Figure 2A). The volcano plot represents dysregulated tissue metabolites between the EC and CO groups (an unpaired *t*-test (with a *p*-value ≤ 0.05) and a fold change (FC) cutoff of 2). This analysis revealed 99 significantly dysregulated metabolites in the EC group compared to the CO group. Specifically, 40 metabolites were upregulated (indicated in red), while 59 metabolites were downregulated (indicated in blue). 

### 3.4. Metabolomic Profiling Between HY and CO

**Figure 3:**
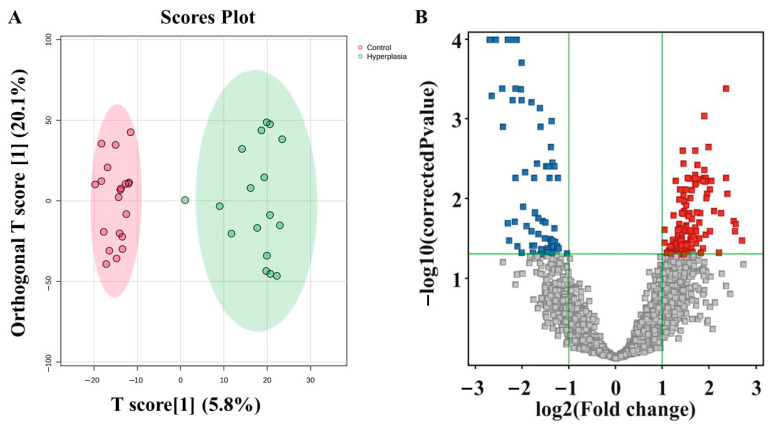
Metabolomic profiling of Hyperplasia vs. Control groups. (**A**) The OPLS-DA score plot illustrates the clear separation of metabolomic profiles between the hyperplasia and control groups. The robust model, validated with a larger dataset (n = 100), demonstrates strong fitness (R2Y = 0.938) and predictive ability (Q2 =0.63). (**B**) The volcano plot highlights significantly dysregulated metabolites in the hyperplasia group compared to the control.

Figure 3 represents the metabolites that distinguished between HY and CO. OPLS-DA, a supervised multivariate approach, is shown in Figure 3A. The distinct separation of the HY group from CO suggests that tissue metabolites may be useful for identifying HY. An unpaired *t*-test (*p*-value < 0.05) and fold change (FC cutoff of 2.0) were used to analyze the volcano plot between the HY and CO groups. The volcano plot shows 195 significantly dysregulated metabolites in HY compared to CO, where 130 and 65 metabolites were up- (red) and downregulated (blue) (Figure 3B).

### 3.5. Metabolomic Profiling Between EC and HY

**Figure 4:**
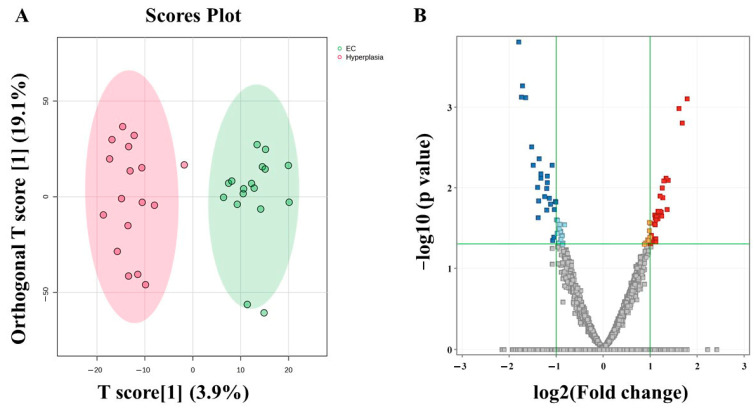
Comparison of metabolomics profiles in endometrial cancer (EC) vs. Hyperplasia (**A**) An orthogonal partial least squares discriminant analysis (OPLS-DA) score plot demonstrates the clear separation of the endometrial cancer (EC) and Hyperplasia groups. The model’s robustness is shown using a larger dataset (n = 100), excellent fit (R2Y = 0.917) and good predictive ability (Q2 = 0.365). (**B**) The volcano plot represents the significant metabolic alterations between the EC and Hyperplasia groups.

The possible biomarkers that changed between EC and HY are displayed in Figure 4. OPLS-DA, a multivariate supervised approach, is shown in Figure 4A. A few metabolites in the EC group are distinct from those in HY, suggesting that tissue metabolomics may be an effective method for distinguishing EC from HY. Between EC and HY, a cross-validated R2Y and Q2 coefficient was noted. A volcano plot analysis was conducted to compare the EC and HY groups using a fold change criterion of 2.0 and a moderate *t*-test (*p*-value < 0.05). The volcano plot shows 57 significantly dysregulated metabolites in EC compared to HY, where 32 and 25 metabolites were up- (red) and downregulated (blue) (Figure 4B).

### 3.6. Evaluation of Metabolite Biomarkers Between EC and CO Groups and Network Pathway

**Figure 5:**
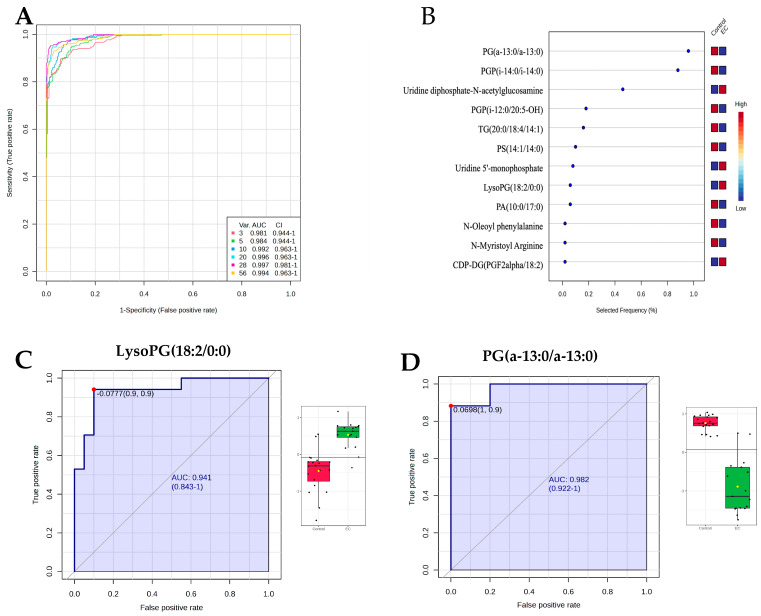
(**A**). Receiver Operating Characteristics (ROC) curve between EC vs. Control. (**B**) The frequency plot shows the significantly dysregulated endogenous metabolites between EC vs. Control. (**C**) The ROC curve of individual biomarker LysoPG(18:2/0:0) (AUC = 0.941) was upregulated. (**D**) PG(a-13:0/a-13:0) (AUC = 0.982) was downregulated in EC compared to Control. The red line indicates the optimal cutoff point for a biomarker, maximizing both sensitivity and specificity.

**Figure 6:**
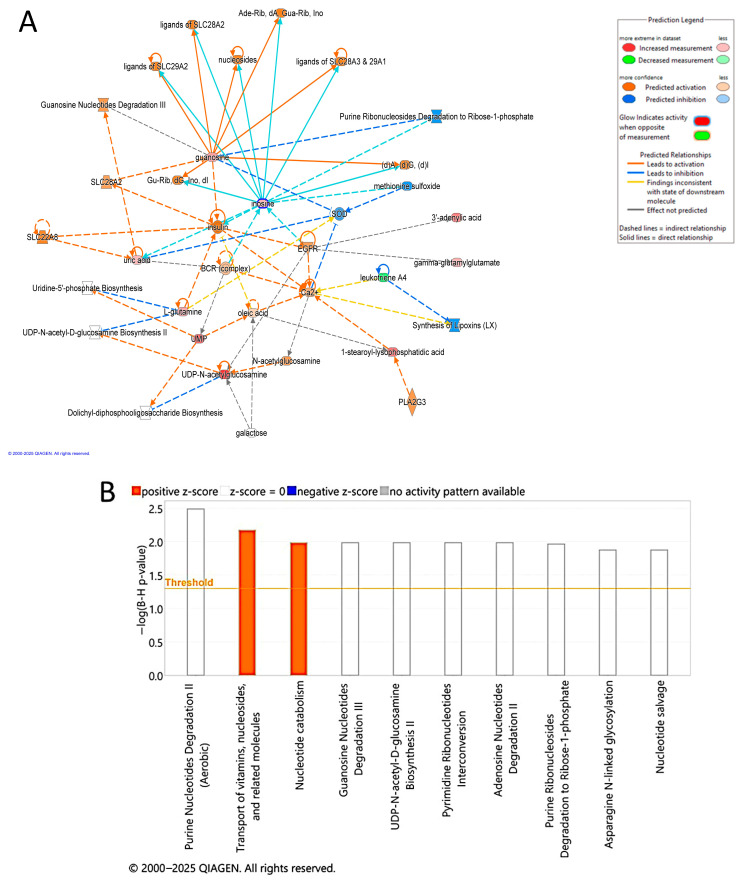
Schematic representation of the highest-scoring network pathways depicting the involvement of the differentially regulated metabolites (**A**) between patients with EC and Control. Nodes colored blue represent downregulation and orange represents upregulation. (**B**) The top canonical pathways generated through IPA (QIAGEN Inc., Hilden, Germany), ranked by the *p*-values. The −log (B-H) *p*-value threshold of 1.4 is equivalent to a Benjamini–Hochberg-corrected *p*-value 0.04.

Multivariate exploratory ROC analysis, utilizing OPLS-DA for feature ranking and classification, was performed on the significantly dysregulated metabolites shared between the EC and CO groups. The analysis of the top 10 metabolites revealed a strong discriminatory ability, with an exploratory ROC curve showing an AUC of 0.992 (Figure 5A). A frequency plot further illustrates the significant dysregulation of endogenous metabolites between the EC and CO groups. The OPLS-DA analysis was carried out to classify and rank features for multivariate exploratory ROC analysis. This was based on the common and significantly dysregulated metabolites identified when comparing the EC and CO groups (Figure 5B). The metabolite LysoPG (18:2/0:0) was upregulated in the EC group and demonstrated an AUC of 0.941 (Figure 5C). Conversely, the PG(a-13:0/a-13:0) metabolite was downregulated in the EC group and showed an AUC of 0.982 (Figure 5D). In these plots, the green box indicates the EC group, while the red box represents the CO group. These differences are statistically significant, with an FDR *p*-value of ≤0.05 and a fold change of ≥1.5. Additionally, the IPA identified cell-to-cell signaling and interaction, skeletal and muscular system development and function as the network pathways affected with the highest score between the EC and CO groups (score of 30) (Figure 6A,B). The top canonical pathways included transport of vitamins, nucleosides and related molecules and nucleotide catabolism.

### 3.7. Evaluation of Metabolite Biomarkers Between HY and CO Groups and Network Pathway

**Figure 7:**
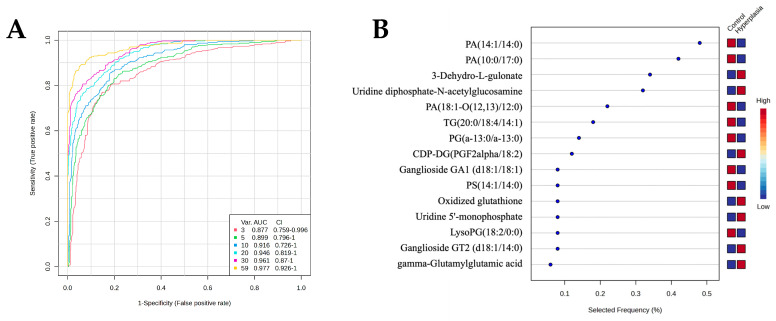

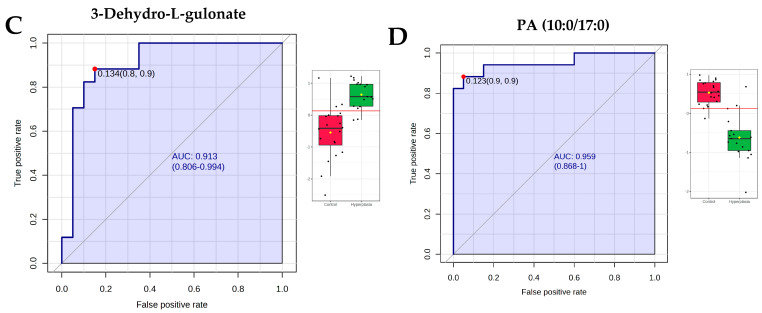
(**A**) The Receiver Operating Characteristics (ROC) curve between the Hyperplasia and Control groups. (**B**) Frequency plot shows the significantly dysregulated endogenous metabolites between Hyperplasia and Control groups. (**C**) ROC curve of individual biomarker 3-Dehydro-L-gulonate (AUC = 0.913) was upregulated, and (**D**) PA (10:0/17:0), (AUC = 0.959) was downregulated in Hyperplasia compared to Control. The red line indicates the optimal cutoff point for a biomarker, maximizing both sensitivity and specificity.

**Figure 8:**
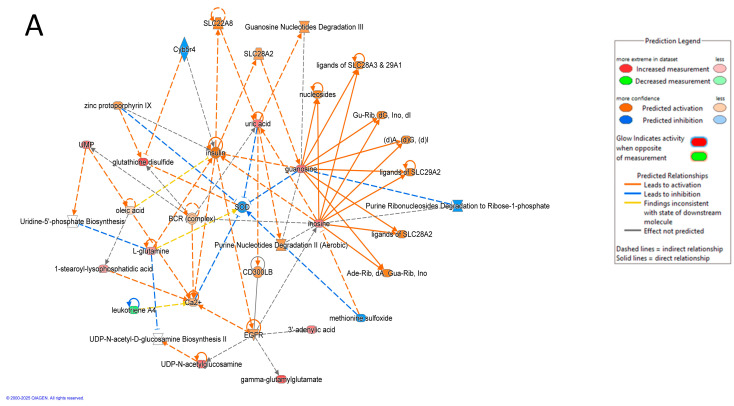

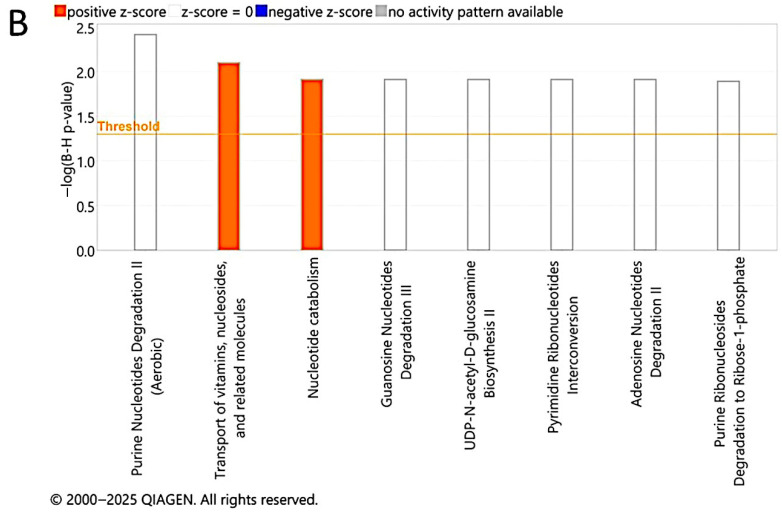
(**A**) Schematic of top network pathways from differentially regulated metabolites between patients with Hyperplasia and Control. Nodes colored blue represent downregulation and orange represents upregulation. (**B**) IPA (QIAGEN Inc., Hilden, Germany) identified and ranked the most significant canonical pathways by *p*-value, from which interaction networks were generated.

A multivariate exploratory ROC analysis was conducted using OPLS-DA as a feature-ranking and classification approach based on the identified common and significantly dysregulated metabolites between the HY and CO groups (Figure 7B). The AUC of the exploratory ROC curve for the top 10 metabolites was 0.916, as shown in Figure 7A. The frequency plot shows the significantly dysregulated endogenous metabolites between HY and CO groups. OPLS-DA was used as a classification and feature-ranking approach for the multivariate exploratory ROC analysis based on the common and significantly dysregulated metabolites identified between the HY and CO groups (Figure 7B).Of the top 15 metabolites, 2 metabolites, 3-Dehyro-L-gluconate (AUC = 0.913), whose expression was upregulated, and PA (10:0/17:0) (AUC = 0.959), whose expression was downregulated in the HY group, are shown in Figure 7C,D. Box and whisker plots for 3-Dehyro-L-gluconate are shown in Figure 7C. The green box indicates the HY groups, and the red box indicates the CO group, with an FDR *p* ≤ 0.05 and a fold change ≥1.5. Box, and whisker plots for PA (10:0/17:0) are shown in Figure 7D, with a fold change ≥1.5 and FDR *p* ≤ 0.05; the green box denotes the HY group, and the red box denotes the CO group. Additionally, the IPA identified inflammatory disease, inflammatory response, and organismal injury as the network pathways affected with the highest score between HY and CO groups (score of 35) (Figure 8A,B). The top canonical pathways included transport of vitamins, nucleosides and related molecules and nucleotide catabolism.

### 3.8. Evaluation of Metabolite Biomarkers Between EC and HY Groups and Network Pathway

A multivariate exploratory ROC analysis was conducted using OPLS-DA as a feature-ranking and classification approach based on the identified common and significantly dysregulated metabolites between the EC and HY groups. The AUC of the exploratory ROC curve for the top 10 metabolites was 0.969, as shown in Supplementary Materials S2, Figure S3A. The frequency plot shows the significantly dysregulated endogenous metabolites between EC and HY groups. OPLS-DA was used as a classification and feature-ranking approach for the multivariate exploratory ROC analysis based on the common and significantly dysregulated metabolites identified between the EC and HY groups (Supplementary Materials S2, Figure S3B). Of the top 8 metabolites, 2 metabolites, PG(a-13:0/a-13:0) (AUC = 0.875), whose expression was downregulated, and PA (8:0/14:0) (AUC = 0.798), whose expression was upregulated in EC group, are shown in Supplementary Materials S2, Figure S3C,D. Box and whisker plots for PG(a-13:0/a-13:0) are shown in Supplementary Materials S2, Figure S3C. The green box indicates the HY group, and the red box indicates the EC group, with an FDR *p* ≤ 0.05 and a fold change ≥1.5. Box and whisker plots for PA (8:0/14:0) are shown in Supplementary Materials S2, Figure S3D, with a fold change ≥1.5 and FDR *p* ≤ 0.05; the green box denotes the HY group, and the red box denotes the EC group. The overlap of the significantly dysregulated endogenous metabolites identified between the three groups is shown in Figure S4.

### 3.9. Pathway Analysis of Significantly Dysregulated Metabolites in EC Compared to HY and CO Groups

**Figure 9:**
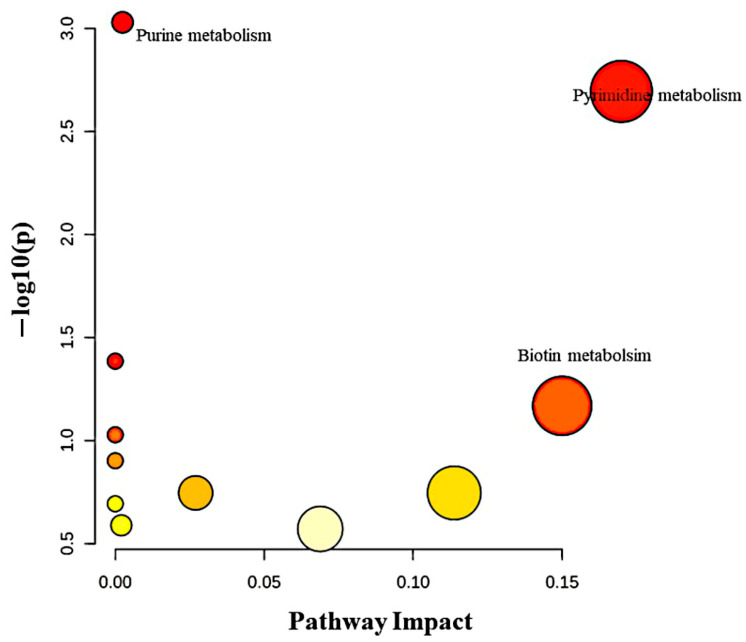
Metabolic pathway analysis of differentially regulated metabolites across the endometrial cancer (EC), Hyperplasia, and Control groups. The intensity of the color (from yellow to red) indicates the statistical significance of the pathway and the size of the circle indicates the number of metabolites enriched within the metabolic pathway.

The pathway analysis of significantly dysregulated metabolites in EC compared to HY and CO is presented in Figure 9. The dysregulated metabolites were involved in metabolic pathways such as pyrimidine, purine and biotin metabolism. A number of metabolites showed a linear change from CO to HY and to EC. These included oxidized glutathione, inosine, icosa-5,8,10,12,14-pentaenoyl-CoA, 3-Methylnonanedioyl-CoA, PG(a-13:0/a-13:0), PS(14:1/14:0), PA (15:0/20:4), PGP(i-14:0/i-14:0), PGP(i-12:0/20:5-OH(18R)), and LysoPG (18:2/0:0) as detailed in Table S5.

## 4. Discussion

In the present study, we characterized the metabolomic changes at different stages of cancer. Cancer progression occurs through different stages, starting with a precancerous stage, followed by cancerous stage, and then metastasis. EC and HY are considered part of a continuum with hyperplasia, often considered a precursor lesion to EC [17]. Both conditions are known to exhibit alterations in cellular proliferation and glandular architecture, along with molecular differences in the tissue architecture that distinguishes hyperplasia from malignant transformation. The changes within the tumor micro-environment (TME) and its related metabolism influence the progression of the neoplastic changes occurring with HY towards EC. Among the key factors shaping the TME in EC are oxidative stress and hypoxia, which are known to play pivotal roles, influencing tumor progression, immune evasion, angiogenesis, and response to therapy. The metabolomic profiling of the endometrial tissue identified significant changes in the levels of 63 endogenous metabolites between the EC and Hy, with the majority of them belonging to lipids, followed by nucleotides and amino acids, indicating metabolic rewiring in these conditions compared to CO.

Lipids are complex biomolecules integral to biological membranes, cell signaling, and energy provision. Cancer is frequently characterized by altered lipid metabolism, with increased lipid synthesis or uptake supporting the rapid growth of cancer cells and the formation of tumors [18,19,20]. A number of different PA lipid species were significantly decreased in EC and HY compared to CO, with a greater decrease in abundance in EC. Phosphatidic acid (PA) is a critical precursor for phospholipid biosynthesis and a known signaling metabolite regulating cell growth and cell membrane dynamics. PA is essential for membrane biogenesis and acts as a precursor for diacylglycerol (DAG) and CDP-DG, both of which are involved in phospholipid metabolism. It plays a central role in lipid signaling, and contributes to the biosynthesis of complex glycerolipids (phosphatidylinositols (PtdIns), phosphatidylserines (PSs), phosphatidylglycerols (PGs) and phosphatidylcholines (PCs)), which contribute to tumor progression by sustaining proliferative signals and inhibiting cell death pathways [19,21,22,23]. Additionally, PA is a key regulator of mTOR signaling, a pathway frequently activated in EC, which may contribute to altered lipid metabolism and cancer progression [24]. The reduction in phosphatidic acid (PA) species, including PA (14:1/14:0), PA (10:0/17:0), PA (15:0, 20:4) and PA (18:1-O(12,13)/12:0), in our cohort suggests impaired lipid biosynthesis and turnover in hyperplastic tissues. A significant decrease was observed in PA (10:0/17:0) in both EC and HY and PA (14:1/14:0) in HY, a critical intermediate in lipid biosynthesis and signal transduction. The reduction in PA levels suggests a possible increase in its utilization in downstream metabolic pathways, including DAG and CDP–DAG synthesis, which are known to support rapid membrane synthesis in tumor cells. The levels of triglyceride (TG) containing long-chain FA (TG(20:0/18:4/14:1)) were lower in EC and HY compared to CO and were also lower in EC when compared to HY. The decrease in TG levels has been noted in previous studies that looked at patients with colorectal cancer [25,26]. Triglycerides are typically stored as lipid droplets and mobilized during periods of increased metabolic demand. The reduction in TG levels in hyperplasia suggests increased lipid consumption or impaired lipid storage, possibly to support the biosynthetic needs of proliferating cells. This decrease in triglycerides reflects altered lipid storage and energy homeostasis.

PA is also known to generate CDP–DAG by condensing with the neucleotide cytidine triphosphate [19]. An increase in the level of CDP-DG(PGF2alpha/18:2) was noted in the EC and HY groups, with higher levels in HY compared to EC. CDP-diacylglycerol (CDP-DG) serves as a precursor for phospholipid biosynthesis and prostaglandin signaling. CDP-DG is a precursor for the synthesis of phosphatidylinositol (PI) and cardiolipin, both of which are critical for cellular signaling and mitochondrial function. The presence of prostaglandin F2alpha (PGF2alpha) in CDP-DG suggests an interaction between lipid metabolism and inflammatory pathways. PGF2 alpha is a known pro-inflammatory eicosanoid that promotes tumor growth, angiogenesis, and immune evasion in EC. Its association with CDP-DG suggests that cancer cells exploit lipid remodeling to sustain chronic inflammation, a hallmark of tumor progression [24]. The decline in ganglioside GA1 (d18:1/18:1) further indicates disruptions in glycosphingolipid metabolism and lipid-mediated signaling. Gangliosides play essential roles in cell–cell interactions, receptor modulation, and immune recognition. The reduction in GA1 suggests impaired glycosphingolipid biosynthesis, potentially altering membrane dynamics and intracellular signaling cascades that regulate cell proliferation.

We also noted a decline in phosphatidylglycerol (PG) levels, particularly PG(a-13:0/a-13:0) in both the EC and HY groups, and in the levels of phosphoglycerophosphated (PGP(i-14:0/i-14:0) and PGP(i-12:0/20:5-OH) in EC compared to CO. The decrease in their levels further supports the reduced phospholipid turnover in cases of cancer and in the precancerous stage. As PG and PGP are precursors for cardiolipin, a key phospholipid in the inner mitochondrial membrane that maintains mitochondrial structure and function, specifically mitochondrial lipid biosynthesis, the decrease likely affects mitochondrial membrane integrity. Its reduction may suggest mitochondrial dysfunction in hyperplasia, which is a characteristic of metabolic shifts towards glycolysis and aligns with mitochondrial stress observed in pre-malignant tissues, which contributes to genomic instability and progression to malignancy. Cardiolipin is essential for oxidative phosphorylation, and its dysregulation is associated with increased glycolysis, a hallmark of cancer metabolism. The reduction in PGP levels indicates that EC cells may be undergoing metabolic reprogramming, shifting from oxidative phosphorylation toward glycolysis to meet their energetic and biosynthetic demands.

Oxidative stress leads to the breakdown of polyunsaturated fatty acids, reducing overall lipid availability. Cancer cells exploit lipids for energy production via beta-oxidation, reducing lipid stores. Decreased phospholipid levels, particularly PE and phosphatidylserines (PS), are linked to hypoxia-induced cellular adaptations. Similarly, the decrease in PS(14:1/14:0) levels suggests alterations in mitochondrial lipid metabolism and apoptotic signaling [27]. PS is a key phospholipid involved in cellular signaling and is a precursor for phosphatidylethanolamine (PE), which is essential for mitochondrial function. Reduced PS levels may reflect an increased conversion to PE, promoting mitochondrial adaptation to support cancer cell survival. Furthermore, PS is known to play a role in apoptotic cell recognition, as externalization of PS on the cell membrane surface serves as a signal for immune clearance [28]. A decrease in the lipid levels in the EC and HY groups can be attributed to increased lipid peroxidation, altered lipid synthesis, enhanced lipid utilization and membrane remodeling. The deregulation of fatty acid (FA) metabolism plays a crucial role in cancer by activating oncogenic signaling pathways and modifying lipid membrane characteristics (composition and saturation). These changes can affect how cancer cells withstand reactive oxygen species (ROS), ultimately influencing their survival and ability to invade and metastasize [29]. The reduction in PS may contribute to immune evasion by EC cells, preventing apoptosis-mediated clearance [30], allowing hyperplastic cells to persist and proliferate unchecked.

Tumors share a common phenotype of uncontrolled cell proliferation and for this they must efficiently generate energy and biomass components in order to expand and disseminate. The required changes in metabolic phenotype are directly driven by oncogene activation and loss of tumor suppressors, and by the constraints imposed by the hypoxic environment of TME. In agreement with this scenario, the lysophosphoglyceride (LysoPG) levels were higher in the tissue of patients with EC, and lower in those with HY compared to the CO group. It can thus serve as a potential biomarker for detection of progression to cancer and as a target of therapy. In contrast to these reductions, the LysoPG (18:2/0:0) levels were significantly elevated, suggesting increased phospholipid hydrolysis. These are active biological amines, or bioactive lipids with signaling activity that are involved in inflammatory signaling, cell proliferation, and tumorigenesis [31,32,33]. Elevated LysoPG levels have been linked to cancer-associated inflammation and immune modulation, processes that facilitate tumor progression [34]. To sustain their proliferation and survival, hypoxic cells display increased uptake of exogenous lysophospholipids, such as lysophosphatidylcholine (LPC) [35]. LysoPG levels were noted to be increased in the ascetic fluid from patients with gastric cancer [36].

But beyond the role of supplying energy, FAs are also involved in other processes, including evading the immune response, angiogenesis, progression of tumors and evading oxidative stress. A key mechanism in evading the immune response and reducing oxidative stress is to reduce the levels of membrane phospholipids. Membrane phospholipids are prone to the attack by reactive oxygen species (ROS), which, in turn, could lead to the initiation of apoptosis, which will ultimately lead to death of the tumor cells [37,38]. In line with this, we found the number of phospholipids, namely, PG and PA, to be decreased in EC and in hyperplasia compared to the CO.

Another significant metabolite that was identified was 3-Dehydro-L-gulonate, a metabolite of the uronic acid pathway, that was increased in both EC and HY compared to CO, with elevated levels in EC. The uronic acid pathway is an alternative pathway for glucose metabolism and provides the substrates for the pentose and glucuronidation pathways. The increase in 3-Dehydro-L-gulonate suggests enhanced oxidative stress and an upregulated pentose phosphate pathway (PPP), which is upregulated to counteract reactive oxygen species (ROS). Accumulation of 3-Dehydro-L-gulonate in hyperplasia may indicate an adaptive response to increased oxidative stress, helping to maintain redox balance and protect cells from oxidative damage that could lead to malignant transformation. The increase in the uronic acid pathway is paralleled with an increase in Uridine diphosphate-N-acetylglucosamine (UDP-GlcNAc), a metabolite of the hexosamine biosynthetic pathway, in EC and HY, with higher levels in EC. UDP-N acetylglucosamine is an intermediate required for N glycosylation, and the folding and secretion of ECM proteins to maintain proteostasis and modulate the extracellular matrix. Remodeling of the extracellular matrix and an increase in its plasticity promote metastatic changes. In a study, it was found that targeting the uronic pathway reduces metastatic progression in lung adenocarcinoma [39,40]. Moreover, the glycosylation of cellular membrane proteins by UDP-GlcNAc impacts the activity of oncogenes, and leads to the activation of oncogenic signaling pathways and metabolic pathways, such as the pentose phosphate pathway [41]. UDP-GlcNAc serves as a donor substrate for O-linked and N-linked glycosylation of proteins, processes that regulate cell proliferation, differentiation, and stress response. The increased UDP-GlcNAc levels in hyperplasia reflect a metabolic adaptation to enhanced glucose flux, as hyperplastic tissue relies on glycolysis rather than oxidative phosphorylation for energy production.

A differential regulation for other nucleic acids was noted between EC and HY. Other significant nucleic acids identified were Uridine 5′-monophosphate, Inosine, guanosine and uric acid, which were increased in EC and HY compared to CO, with higher levels in HY, suggesting increased biosynthetic activity required for rapid proliferation, immune evasion, and altered cellular signaling. Nucleotide metabolism dysregulation, particularly increased UMP levels, suggests an upregulated pyrimidine biosynthetic pathway supporting DNA/RNA synthesis and transcriptional activity in tumor cells. UMP’s role as a precursor for uridine triphosphate (UTP) further reinforces its significance in EC progression. Similarly, elevated inosine and guanosine levels indicate increased purine metabolism, facilitating sustained nucleotide turnover and energy production. Inosine’s role in extracellular signaling through adenosine receptors contributes to an immunosuppressive microenvironment, while guanosine supports oncogenic GTP-binding proteins such as Ras and Rho-family kinases, promoting proliferation and metastasis.

An increase in the levels of gamma-glutamyl glutamic acid, glutamine, and oxidized glutathione was seen in both EC and HY. In a study by Błachnio-Zabielska, a significant increase in oxidative stress levels and a significant decrease in total antioxidant capacity within endometrial cancer tissues compared to the normal endometrium of the control group were observed [38]. Gamma-glutamyl glutamic acid, glutamine, and oxidized glutathione are key intermediates of the gamma glutamyl cycle involved in the regulation of oxidative stress and glutathione metabolism, particularly in glutathione biosynthesis and amino acid transport. In our data, we found an increase in the level of gamma-glutamyl glutamic acid and oxidized glutathione, which indicates increased glutamate metabolism and glutathione turnover. GSH is a major antioxidant that protects cells from oxidative damage. Elevated gamma-glutamyl glutamic acid reflects enhanced activity of the gamma-glutamyl cycle, which is crucial for glutathione biosynthesis and detoxification, indicating a cellular response to increased oxidative stress in both EC and hyperplasia. In EC and HY, the increased levels of gamma-glutamyl glutamic acid, glutamine, and oxidized glutathione indicate a metabolic shift toward supporting oxidative stress adaptation. Glutamine serves as a crucial metabolic fuel, supplying carbon to the tricarboxylic acid cycle for generation of energy, and providing precursors for nucleotide and lipid biosynthesis. Its increased availability suggests an upregulation of glutaminolysis, which facilitates rapid cell division and sustains biosynthetic processes necessary for tumor growth. This supports cancer cell survival by enhancing detoxification mechanisms and protecting against oxidative damage induced by rapid proliferation.

A biomarker analysis using ROC analysis was carried out to identify the significant metabolites in EC and HY. The top metabolites identified that can discriminate EC and HY from CO were identified. This revealed a decrease in the levels of the lipid species PA (14:1/14:0), PA (10:0/17:0), PA (18:1-O(12,13)/12:0), PG(a-13:0/a-13:0),Ganglioside GA1 (d18:1/18:1), PS(14:1/14:0), TG(20:0/18:4/14:1), CDP-DG(PGF2alpha/18:2), while there was an increase in the levels of 3-Dehydro-L-gulonate, Uridine diphosphate-N-acetylglucosamine, Ganglioside GT2(d18:1/14:0), and gamma-glutamyl glutamic acid in cases of hyperplasia as compared to CO. Bioinformatics and network pathway analysis showed the differential impact of the altered metabolites in both stages. IPA identified the pathway related to cell-to-cell signaling and interaction, skeletal and muscular system development and function, and small-molecule biochemistry with the highest score in cases of EC, while the pathway related to inflammatory disease, inflammatory response, organismal injury and abnormalities had the highest scores in cases of HY. The network pathways of both HY and EC centered around regulation of epidermal growth factor receptor [EGFR], insulin and superoxide dismutase (oxidative stress)-related signaling, although the increase in gamma glutamylglutamate and glutathione disulfide was noted in cases of HY. EGFR signaling is known to drive dysregulated FA metabolism, and influence lipid membrane composition and saturation to modulate tolerance to reactive oxygen species (ROS) and cancer cell survival [42].

The tumor microenvironment and immune landscape change significantly during the transition from hyperplasia to EC. Hyperplasia remains largely immunologically reactive, allowing immune cells to surveil and potentially eliminate abnormal cells. As hyperplasia is characterized by excessive proliferation of the endometrial lining, the changes in the metabolite levels reflect shifts in lipid metabolism, nucleotide biosynthesis, oxidative stress response, and glycosylation pathways. Lipids are important for sustaining tumorigenesis in [19]; in this context, the decrease in the levels of phospholipids and triglycerides, alongside an increase in oxidative-stress-related and glycan metabolism intermediates, was noted in HY, indicating metabolic reprogramming that supports cell proliferation and survival. However, EC progressively develops mechanisms for immune evasion and creating a tumor-supportive microenvironment. These features are absent or minimal in hyperplasia, further supporting the distinction between benign and malignant endometrial conditions.

Early detection of cancer is crucial because it allows for prompt treatment, which can help prevent the disease from worsening and spreading. While many biomarkers from various sources like lipids, sugars, nucleic acids, and proteins have been investigated, only a handful, such as CA-125 or HE4, have made it into routine clinical use [43,44]. So, it is important to develop safer, non-invasive and more cost-effective alternative standard diagnostic practices. Our metabolomic study offers novel insights into endometrial hyperplasia, EC, and controls, although it does have limitations. Specifically, the relatively small number of observations might restrict the generalizability of our findings. The use of multivariate ROC analysis in a limited cohort may also increase the risk of bias due to the small number of observations, increase the probability of finding a random association between a given metabolite and the outcome due to total number of comparison overfitting, and may overstate discriminatory performance. Our findings are exploratory and need validation in larger, independent studies.

## 5. Conclusions

Endometrial cancer and HY exhibit distinct metabolic profiles, reflecting differences in cellular proliferation, lipid remodeling, and metabolic stress adaptation. Patients with HY, a precursor to EC, demonstrate elevated levels of key phospholipids, triglycerides, gangliosides, and oxidative stress markers, while EC is characterized by enhanced inflammatory lipid signaling and nucleotide metabolism. This exhibits distinguishing features in tumor cellular metabolism that collectively enhance cell development while maintaining redox balance and cellular homeostasis. Development of sensitive and specific biomarkers will help in the diagnosis of EC and HY. This, in turn, will serve to make an informed decision regarding the mode of treatment, specifically whether to rely on hormonal treatment and avoid hysterectomy. To inform treatment decisions in women diagnosed with endometrial hyperplasia, quantification of the potential for concurrent endometrial cancer and the future risk of progression to cancer is required.

## Data Availability

All the data generated or analyzed in the current study are included in this article.

## Supplementary Materials

The following supporting information can be downloaded at: https://www.mdpi.com/article/10.3390/metabo15070458/s1, Supplementary Materials S1; Table S1: Clinico-pathological characteristics of the 54 women enrolled in this study. Table S2: Raw data show mass ion features detected in tissue samples of the EC, Hyperplasia and Control groups. Table S3: Metabolites were significantly dysregulated in the EC, Hyperplasia and Control groups. One-way ANOVA (Tukey’s post hoc, FDR *p* < 0.05). Table S4: Exogenous and endogenous metabolites in tissue samples of the EC, Hyperplasia and Control patients. One-way ANOVA (Tukey’s post hoc, FDR *p* < 0.05). Table S5: Endogenous metabolites in tissue samples of EC, Hyperplasia and Control samples. One-way ANOVA (Tukey’s post hoc, FDR *p* < 0.05). Supplementary Materials S2; Figure S1: Profile of upregulated metabolites in EC compared to Hyperplasia and Control after performing fold change of 2; Figure S2: Profile of 33 downregulated metabolites in EC compared to Hyperplasia and Control after performing fold change of 2; Figure S3. Biomarker evaluation in endometrial cancer (EC) and Hyperplasia; A. The Receiver Operating Characteristics (ROC) curve was generated by the OPLS-DA model, with Area Under the Curve (AUC) values calculated from the combination of 2, 3, 5, 7, 10, and 13 metabolites. B. Frequency plot showing the top 8 significantly dysregulated metabolites in the EC and Hyperplasia groups. ROC curve of individual metabolite biomarkers: C. (PG(a-13:0/a-13:0), with an AUC of 0.875; box plot (*p* ≤ 0.05 and fold change ≥1.5), where red represents EC group and green represents the Hyperplasia group; and D. PA (8:0/14:0), with an AUC of 0.798; box plot (*p* ≤ 0.05 and fold change ≥1.5), where red represents EC group and green represents the Hyperplasia group; Figure S4: Venn diagram of the significantly differentially abundant endogenous metabolite features among three groups (endometrial cancer vs. hyperplasia, endometrial cancer vs. control, and hyperplasia vs. control) as determined by one-way ANOVA with Tukey’s post hoc test, FDR *p* < 0.05. Six metabolites common between the groups.
