# Supplementary material for: Tissue-Based Metabolomic Profiling of Endometrial Cancer and Hyperplasia
Source: Metabolites. 2025 Jul 5;15(7):458. doi: 10.3390/metabo15070458 (PMC12299690; doi:10.3390/metabo15070458)
Supplement: Supplementary file 1 [file metabolites-15-00458-s001.zip › Supplementary Materials Figure S4 - Venn diagram.pdf]

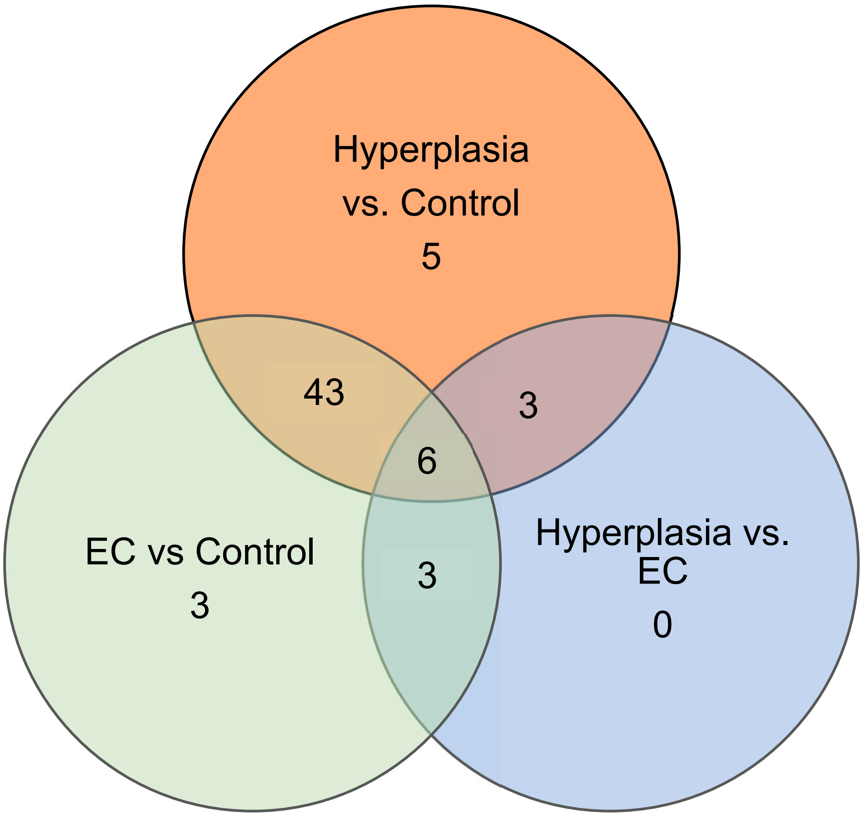

Figure S4 Venn diagram of the significantly differentially abundant endogenous metabolite features among three groups (endometrial cancer vs. Hyperplasia, endometrial cancer vs. Control, and Hyperplasia vs. Control) as determined by one-way ANOVA with Tukey's post-hoc test, FDR $p<0.05$. Six metabolites were common between the three groups, while 3 metabolites were unique in EC vs control, and five metabolites in HY vs controls as shown in Supplementary data S4.
