# Supplementary material for: Tissue-Based Metabolomic Profiling of Endometrial Cancer and Hyperplasia
Source: Metabolites. 2025 Jul 5;15(7):458. doi: 10.3390/metabo15070458 (PMC12299690; doi:10.3390/metabo15070458)

Figure S1: Showing profile of 30 Upregulated metabolites in EC compared to Hyperplasia and Control after performing fold change 2

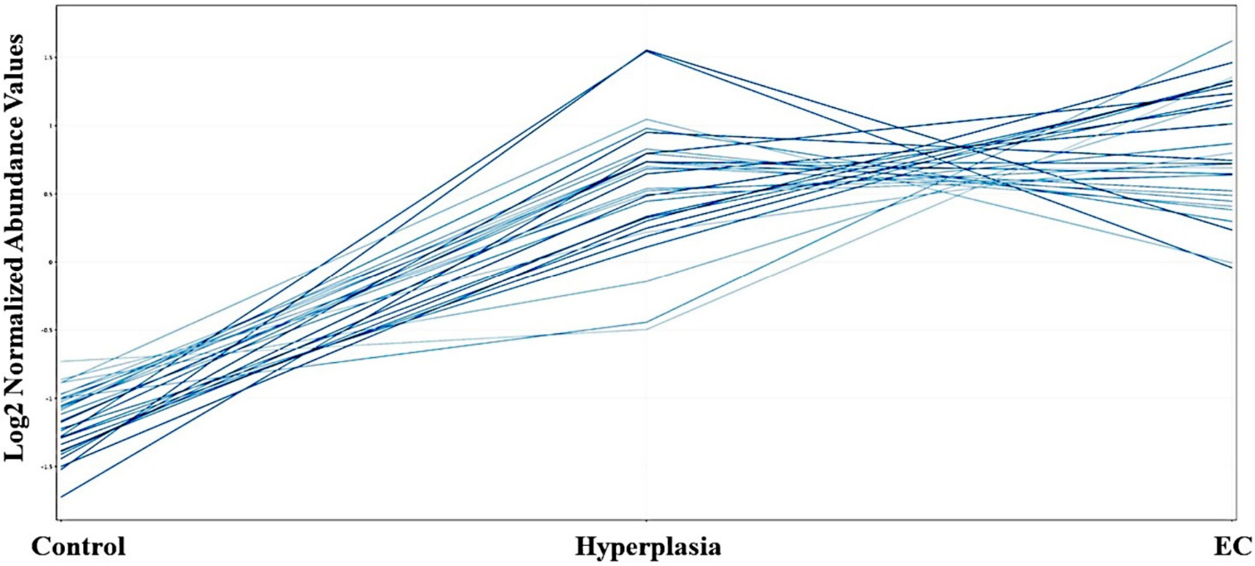

Figure S2: Showing profile of 33 Downregulated metabolites in EC compared to Hyperplasia and Control after performing fold change 2

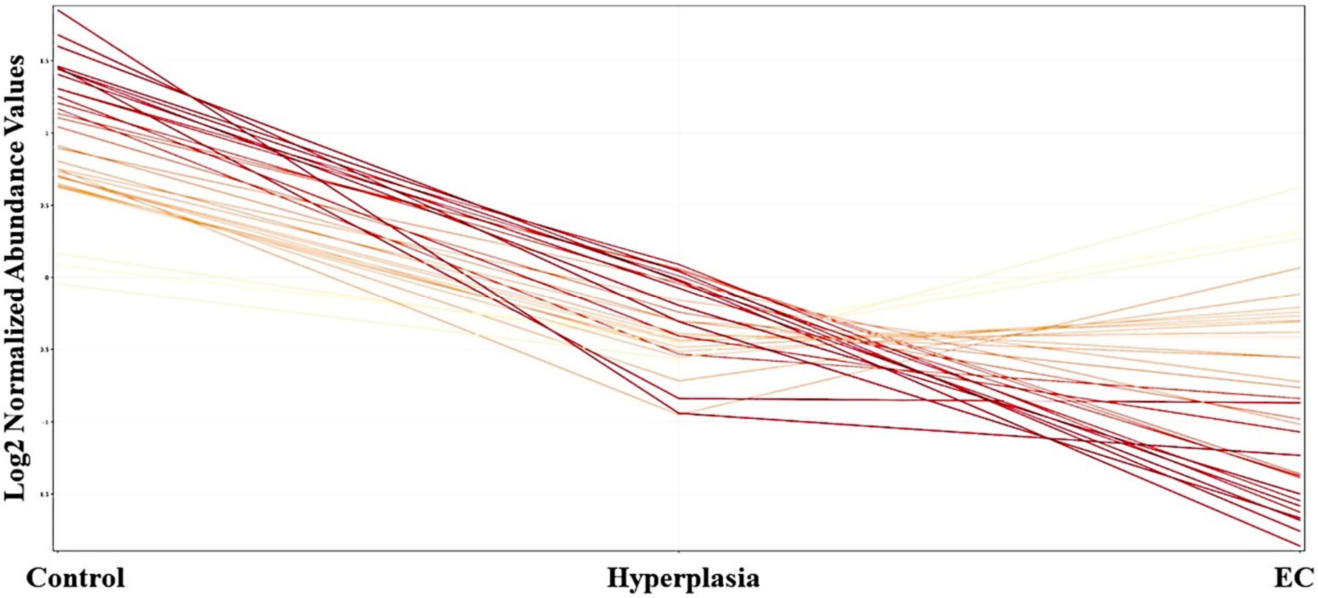

Figure S3. Biomarker evaluation in Endometrial cancer (EC) and Hyperplasia (HY). **A**. The Receiver Operating Characteristics (ROC) curve between EC vs HY **B**. Frequency plot showing significantly dysregulated metabolites in the EC and HY groups. ROC curve of individual metabolite biomarkers: **C**. (**PG(a-13:0/a-13:0)**, with an AUC of 0.875 was upregulated were red represents EC group and green represents the HY group; and **D**. PA (8:0/14:0), with an AUC of 0.798 downregulated in HY compared to EC; where red represents EC group and green represents the HY group. The red line shown in the box plot represents the median concentration for each individual biomarker.

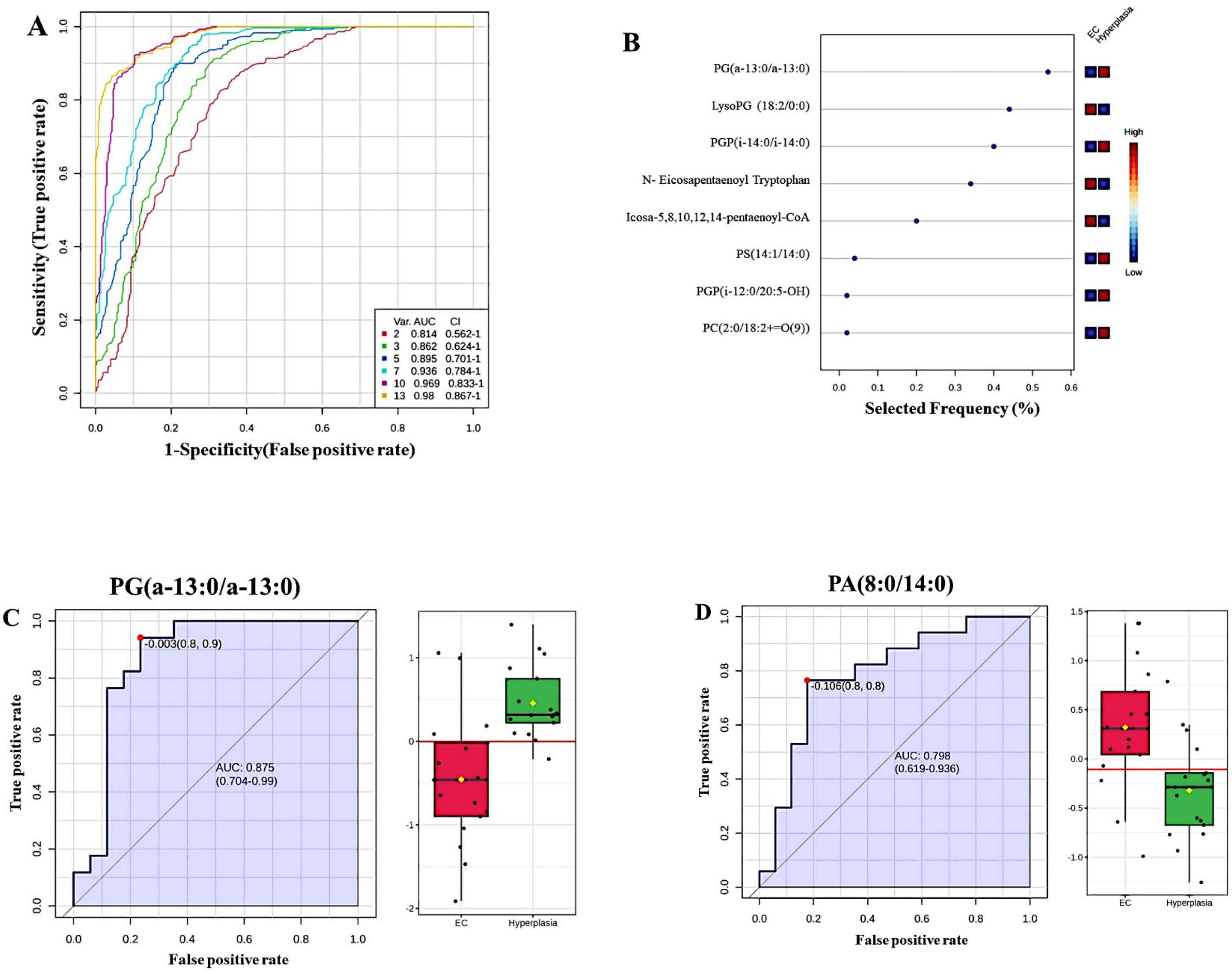

Supplement: Supplementary file 1 [file metabolites-15-00458-s001.zip › Supplementary Materials S2.pdf]
